# Alloying effects on deformation induced microstructure evolution in copper

**DOI:** 10.1038/s41598-024-73926-3

**Published:** 2024-10-13

**Authors:** Reeju Pokharel, Tongjun Niu, Sara Ricci, Bjørn Clausen, Levente Balogh, Lucas Ravkov, Ramon Martinez, Chanho Lee, Sven Vogel, Carl M. Cady, Michael A. Torrez, Benjamin K. Derby, Jonathan G. Gigax, Nicola Bonora, Nan Li, Saryu J. Fensin

**Affiliations:** 1 Materials Science and Technology Division, Los Alamos National Laboratory, Los Alamos, NM 87544 USA; 2 MPA-CINT, Los Alamos National Laboratory, Los Alamos, NM 87544 USA; 3 Department of Civil and Mechanical Engineering, University of Cassino and Southern Lazio, I-03043 Cassino, Italy; 4 Department of Mechanical and Materials Engineering, Queen’s University, Kingston, ON Canada

**Keywords:** Mechanical properties, Metals and alloys

## Abstract

In this work, we investigated the effects of alloying elements on plastic deformation and microstructure evolution in polycrystalline copper (Cu) and Cu alloyed with 1 wt.$\%$ lead (Cu-1$\%$Pb). These materials were selected due to the size mismatch between Cu and Pb, with the latter forming precipitates at grain boundaries. Multi-modal characterization techniques, including neutron diffraction, electron backscatter diffraction (EBSD), and transmission electron microscopy (TEM), along with finite element simulations were employed to study the deformation behavior across multiple length scales. While both Cu and Cu-1$\%$Pb exhibited similar macroscale response and final deformation textures, both dislocation line profile analysis and TEM revealed increased dislocation density in deformed Cu-1$\%$Pb specimens. The presence of lead precipitates also significantly affected local plastic deformation during compression, with their influence diminishing with increasing strain. These results demonstrate the complex relationships between alloying elements, plastic deformation, microstructural evolution, and material behavior under load. The insights gained from this multi-scale and multi-technique approach contribute to the fundamental understanding of microstructural evolution in immiscible alloys and are valuable for tailoring the properties of structural materials for specific engineering applications.

## Introduction

Two-phase metallic systems, characterized by limited or no miscibility of their components in both liquid and solid states even at high temperatures^1^, offer unique properties that can be tailored for specific performance outcomes^2–4^. For example, the copper-tungsten (Cu-W) system combines the high thermo-mechanical stability and radiation shielding capability of tungsten (W) with the superior thermal conductivity and electrical performance of copper (Cu)^5^. Similarly, introducing a small amount of lead (Pb) into a high-purity Cu matrix can enhance the fracture toughness of Cu^6^. However, these immiscible metallic systems also present challenges due to the strong segregation tendencies arising from mutual immiscibility and significant density differences between the components^7^.

Understanding the deformation and fracture mechanisms in immiscible ductile metallic systems is crucial for both materials science and engineering applications. In single-phase and high-purity ductile materials, deformation and fracture typically follow a sequence of void nucleation, growth, and coalescence, with features like grain boundaries, inclusions, vacancies, and microstructural heterogeneities serving as potential sites for initial void nucleation. However, the influence of a hard or soft second phase (e.g., W versus Pb in a Cu matrix) on damage nucleation and subsequent evolution in engineering materials is not fully understood. Previous work on high-purity copper and copper alloyed with 1 wt.$\%$ lead (Cu-1$\%$Pb) under shock loading^6,8^ revealed that Cu-1$\%$Pb exhibits a nucleation-dominated damage evolution with a higher number of smaller voids, while high-purity single-phase Cu shows a growth-dominated mechanism with fewer, larger voids. These variations in material properties and macroscopic mechanical responses are attributed to differences in microstructure, defect content, and their interactions^8–10^. Building upon these findings, the current work investigates the quasi-static deformation behaviors and associated microstructural developments in these materials under uniaxial compression tests.

Various techniques are employed for qualitative and quantitative microstructure characterization. Cross-sectional transmission electron microscopy (TEM)^11,12^ and electron backscatter diffraction (EBSD)^13,14^ offer direct insights into dislocation generation, structuring, and strain fields induced by material plasticity. Complementarily, neutron diffraction serves as a non-destructive method for studying deformation-induced microstructural changes^15^, providing a statistical grain sampling within a polycrystal. Neutron powder diffraction is particularly effective at distinguishing between different phases, crystal orientation distributions, defect densities, temperature effects, and compositional changes in bulk metallic specimens. The peak profile intensity reveals texture evolution, while peak width and profile shape correlate with defect content, microstrains, crystallite size (<1 $\mu$m), dislocation structuring, and arrangements. In cubic materials, plastic deformation typically results from dislocation movement across specific slip systems, causing lattice distortion evident as diffraction peak profile broadening and shape changes^16^. Local heterogeneities leading to microstrain and sub-micron crystallite sizes due to dislocation cell structure formation further contribute to this broadening. Dislocation line profile analysis (DLPA) is an effective method for extracting semi-quantitative information about dislocation density, coherent crystallite size, and dislocation arrangement from both conventional and novel materials such as additively manufactured (AM) and high-entropy alloys^17–20^. Case studies by Ungar et al.^21^ and J. Gubicza et al.^16^ further highlight its utility in analyzing phase transformation, dislocation density, and microstructural stability under severe plastic deformation.

In this study, we examine polycrystalline copper and copper alloyed with 1 wt.$\%$ lead (Cu-1$\%$Pb) to elucidate the relationship between alloying elements, plastic deformation, and microstructural evolution. These model materials are chosen for their ability to provide insights into the influence of a secondary soft phase on mechanical behavior. We employ neutron diffraction to probe texture evolution and defect creation, complemented by EBSD and TEM analyses. Despite Cu and Cu-1$\%$Pb showing similar macroscopic stress-strain responses under compression, the presence of a second phase induces subtle changes at the local microstructural level during deformation. Preliminary micromechanical simulations are conducted to understand the influence of addition of small amount of Pb particles on deformation fields of the Cu matrix. Note that no mechanical mixing of lead was observed at the strain levels investigated in this study. The experimental data obtained here is anticipated to assist in calibrating and validating crystal plasticity models, enhancing the predictability of these microstructure-sensitive models.

## Results

This section presents our findings on the interplay between alloying elements, plastic deformation, and microstructural evolution in polycrystalline Cu and Cu-1$\%$Pb. Initial microstructural characterization revealed right-skewed grain size distributions in both materials. However, for simplicity and quantitative comparison, we applied Gaussian statistics to the data. For pure Cu samples, the calculated mean grain size was 24 $\mu$m with a standard deviation of 30 $\mu$m. The Cu-1$\%$Pb samples had a calculated mean grain size of 21 $\mu$m with a standard deviation of 17 $\mu$m. The large standard deviation in pure Cu, exceeding its mean, reflects the significant variability in grain sizes. SEM analysis indicated homogeneously distributed lead particles within the copper matrix, with particle diameters ranging from 1 to 5 $\mu$m. However, the orientation of these particles could not be indexed through EBSD, TEM, or neutron diffraction techniques. Due to the small size of the Pb particles, which were not fully characterized by the experimental modalities used in this study, we did not monitor two-phase interactions, strain partitioning, or attributes like grain aspect ratio changes with deformation for both phases. These baseline characteristics set the stage for our subsequent detailed measurements and observations of the materials’ behavior under deformation.

**Fig. 1:**
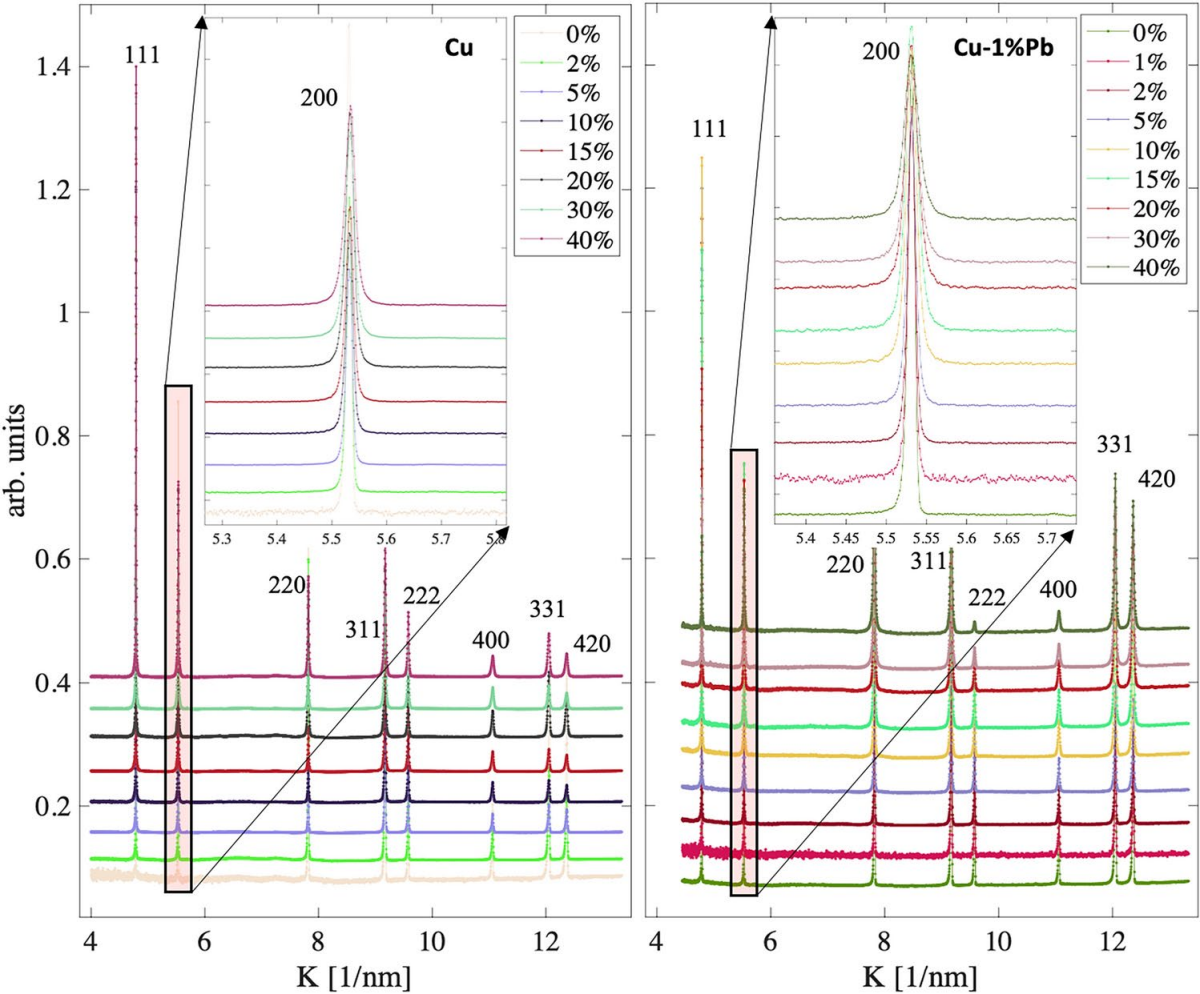
High-resolution neutron diffraction line profile data for copper (Cu) and copper 1 wt.$\%$ lead (Cu-1$\%$Pb). Insets show the 200 reflection, which has the largest dislocation contrast factor for cubic symmetry, illustrating peak broadening due to the applied compressive deformation.

The diffraction patterns obtained at varying strains for both Cu and Cu-1$\%$Pb specimens are shown in Figure 1. The diffraction profile asymmetry visible in Figure 1 corresponds to the characteristic peak shape asymmetry of time-of-flight neutron diffraction measurements. In the diffraction line profile analysis, this asymmetry is accounted for through the calibration/instrumental pattern. Although Cu and Pb have face-centered cubic (fcc) structures, their near-complete immiscibility in the solid state arises from a significant difference in atomic radii (37$\%$)^22^. However, due to the small particle size and minimal Pb content in the compression specimens, only Cu peaks are discernible in the diffraction patterns. Nevertheless, a significant increase in the diffraction peak width is observed in both materials with increasing deformation levels. This broadening is primarily attributed to microstructural changes, such as defect generation, grain refinement, and strain accumulation, which are necessary to accommodate the externally imposed macroscopic load.

**Fig. 2:**
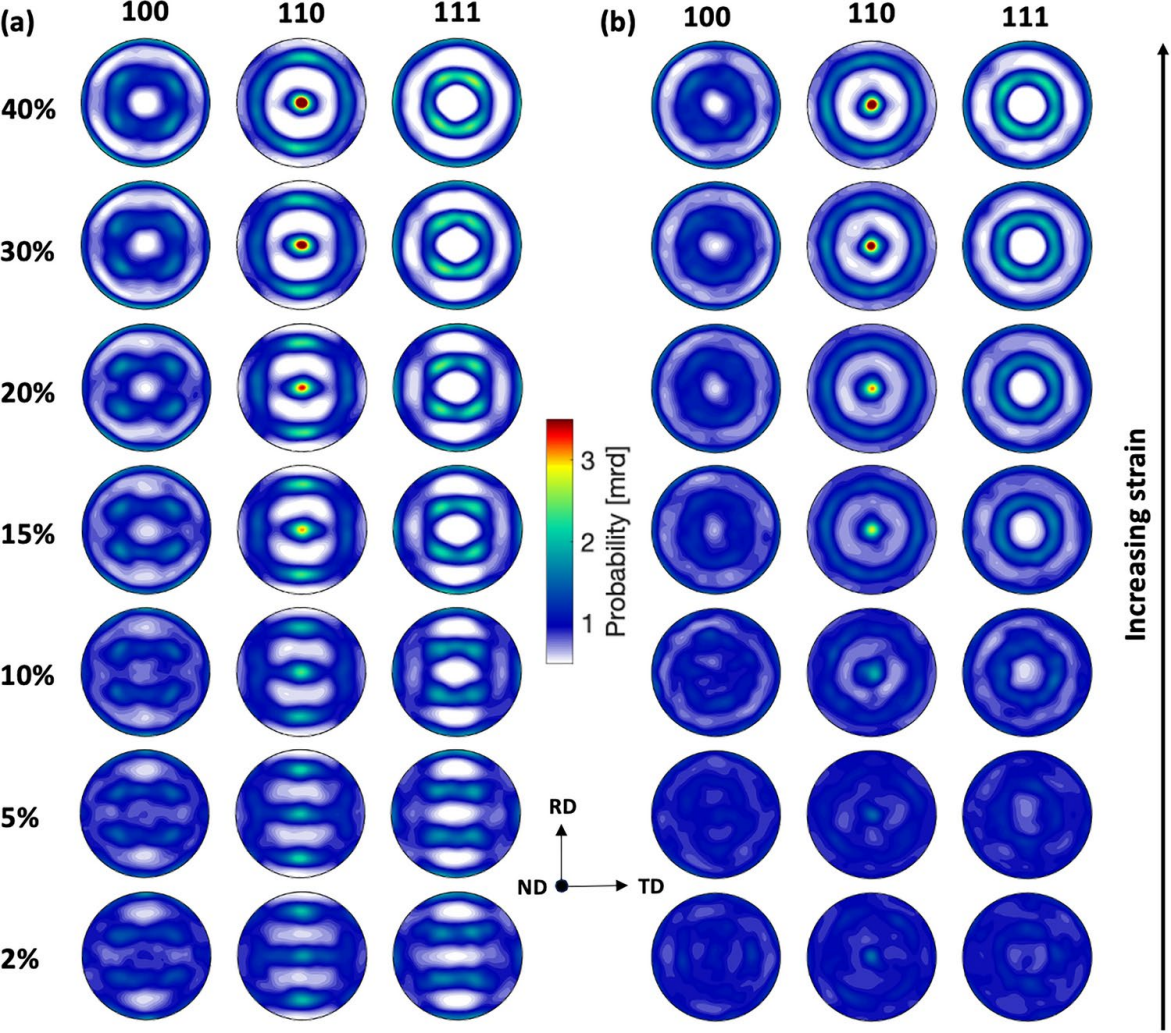
Pole figures illustrating deformation texture evolution due to imposed compressive deformation for (**a**) Cu and (**b**) Cu-1$\%$Pb at different strains. The pole figures are plotted on a common scale ranging from 0.5 to 3.5 mrd. The center of the pole figure is parallel to the compression axis. With increasing strain, both materials show prominent increase in 110 peak intensity, indicating crystals with 110 plane normal orient along the loading direction as deformation progresses.

The texture evolution in Cu and Cu-1$\%$Pb up to a 40$\%$ true strain is shown in Figure 2. Both materials were extracted from rolled plates and subjected to heat treatment after machining the compression specimens. Initially, Cu-1$\%$Pb exhibited a random texture, while the Cu specimen showed a weak remnant of the rolling texture. As a result, minor discrepancies are noticeable in the early deformation textures, which can be attributed to the slightly divergent starting textures between the two materials. Despite the initial texture differences, both materials demonstrate a consistent trend in the development of preferred orientation upon deformation, reaching a peak value of roughly 3.5 multiples of a random distribution (mrd) at 40$\%$ strain. As expected during the compressive loading of fcc metals, a 110 fiber texture develops along the loading axis in both instances, as indicated by the increased intensity with increasing deformation.

**Fig. 3:**
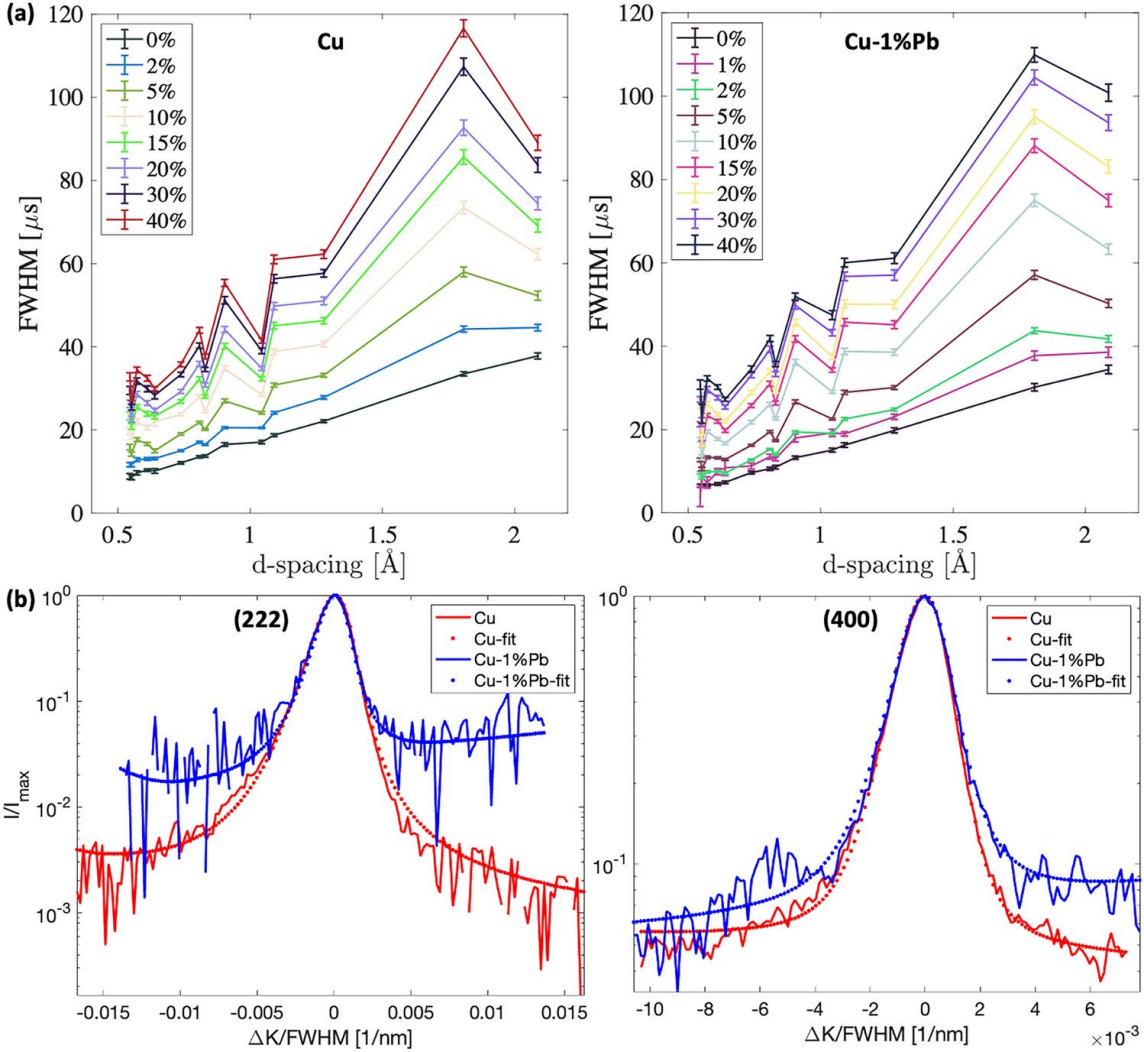
(**a**) Peak width as a function of crystallographic planes and deformation for copper and copper with 1 wt.$\%$ lead. Assuming that dislocations are the primary contributors to the observed broadening, the non-linear increase in width arises from the anisotropic elastic properties of the two materials. (**b**) Peak profiles of the (222) and (400) reflections for Cu (red) and Cu-1$\%$Pb (blue) after 40$\%$ strain. Profiles are background-subtracted, centered, and normalized to both peak maxima and FWHM, with normalized intensities plotted on a logarithmic scale. Solid lines represent raw data, while dotted lines correspond to fits from line profile analysis. Although the FWHMs vary within a narrow range, the tail regions of the peaks from the two materials exhibit significant variations in broadening.

Figure 3(a) shows a Williamson-Hall plot derived from the full width at half maximum (FWHM) of the time-of-flight neutron diffraction data. The FWHM serves as an indicator of ‘microstrain’ or ‘intragranular strain’ resulting from variations in interatomic spacing associated with defects (such as dislocations or other heterogeneities), where K is defined as the reciprocal value of d, K=1/d. The instrumental resolution, determined from the annealed specimen at 0$\%$ strain (assumed to be defect-free), consistently shows an increase in broadening with the scattering angle. The observed anisotropic line broadening at different strains reflects the average dislocation contrast factors in polycrystals, a signature behavior of peak widths of cold-worked metals. This non-linear broadening stems from the anisotropic contrast factor associated with each active crystallographic slip system in a specific crystal structure^16^. The average dislocation contrast factor of an hkl reflection in cubic polycrystals is expressed by Equation 1^23,24^:

1 $$\begin{aligned} C_{hkl} = C_{h00}(1-qH^{2}) \text { and }\nonumber \\ H^{2 } = \frac{h^2k^2+h^2l^2+k^2l^2}{(h^2+k^2+l^2)^2}, \end{aligned}$$

where *q* and $\hbox {C}_{h00}$ are dislocation character parameter and average contrast factor for h00 reflections, respectively. Both depend on the single crystal elastic constants of the material and are determined numerically^24,25^. The systematic increase in broadening with higher-order hkl reflections indicates an increase in dislocation density. As the broadening due to crystallite size remains constant with respect to hkl order when expressed in terms of diffraction vector length, the increase as a function of K is attributed solely to microstrain^26–28^. Additionally, the inhomogeneous distribution of lead inclusions, initially segregated at grain boundaries in the Cu-1$\%$Pb compression specimens^6^, might also contribute to some extent of peak broadening.

To isolate the effects of various microstructural factors on peak broadening, a dislocation line profile analysis (DLPA) was performed. Table 1 summarizes the microstructural information extracted from DLPA using the extended convolution multiple whole profile (eCMWP) software^26^. The dislocation density measurements using DLPA are only reported for samples strained to 5$$\%$$ and beyond, as the undeformed or slightly deformed samples do not exhibit detectable dislocation-related peak broadening, indicating dislocation densities below the neutron diffraction measurement threshold of approximately $$0.1*10^{14}$$$$\hbox {m}^{-2}$$. This analysis yields several microstructure parameters, including the area-weighted mean coherently scattering crystallite size (sub-grain size, $$\chi$$), dislocation density ($$\rho$$), dislocation arrangement ($$M^{*}$$), and the parameter *q* which characterizes the dislocation contrast factors. From this, the fraction of edge and screw dislocations are estimated.

**Table 1:** Summary of the DLPA for Cu and Cu-1$\%$Pb.

Strain [$\%$]	$\rho$ [$\times 10^{14}/\hbox {m}^2$]	$\chi$ [nm]	$\hbox {M}^{*}$	q	Edge / screw fractions [$\%$]
Cu: 5	0.12 (0.03)	186 (1.8)	0.60 (- -)	1.7 (- -)	-
10	0.95 (0.04)	170 (1.3)	0.60 (- -)	1.7 (- -)	-
15	2.02 (0.09)	175 (3.7)	0.66 (1.0)	1.56 (0.05)	70 / 30
20	4.50 (0.12)	171 (2.6)	0.36 (0.22)	1.41 (0.03)	89 / 11
30	5.69 (0.16)	132 (1.8)	0.43 (0.26)	1.88 (0.03)	30 / 70
40	8.03 (0.17)	129 (1.5)	0.43 (0.16)	1.88 (0.02)	30 / 70
Cu-1$\%$Pb: 5	0.36 (0.02)	194 (1.4)	0.59 (- -)	1.7 (- -)	-
10	3.44 (0.06)	156 (0.7)	0.59 (- -)	1.7 (- -)	-
15	7.59 (0.38)	125 (0.7)	0.15 (0.12)	1.85 (0.03)	34 / 66
20	8.97 (0.21)	112 (0.7)	0.17 (0.06)	1.78 (0.03)	43 / 57
30	13.53 (0.26)	98 (0.6)	0.16 (0.04)	1.76 (0.02)	46 / 54
40	14.84 (0.36)	92 (0.7)	0.17 (0.06)	1.72 (0.02)	51 / 49

The dislocation density increases with deformation, reaching values of $$\sim 0.8 \times 10^{15} \hbox {m}^{-2}$$ for Cu and $$\sim 1.4 \times 10^{15} \hbox {m}^{-2}$$ for Cu-1$$\%$$Pb at 40$$\%$$ compressive strain. Due to low dislocation density below 5$$\%$$ strains, the DLPA was not performed. For the 5$$\%$$ and 10$$\%$$ strain data, refinements necessitated additional constraints to ensure convergence. Thus, the dislocation character parameter *q* was fixed at 1.7, suggesting equal proportions of edge and screw dislocations, while the dislocation parameter ($$M^{*}$$) was set to approximately 0.6 for both materials. At 5$$\%$$ strain, both materials exhibited $$\rho$$ values of around $$10^{13}$$$$\hbox {m}^{-2}$$, which is at the detection limit of the neutron diffraction measurements.

The addition of an alloying element to Cu increased the dislocation content with deformation, nearly doubling the number of dislocations generated during deformation at the maximum strain achieved in the experiments. Concurrently, the crystallite size ($\chi$) - representative of the coherently scattering domain size - decreased with increasing deformation, slightly more so in Cu-1$\%$Pb at 40$\%$ compressive strain. Typically, in metals, the coherently scattering crystallite size corresponds to the dislocation cell size within the grains delimited by high-angle boundaries.

A systematically lower $M^{*}$ parameter, related to the dislocation arrangement^29^, was observed in Cu-1$\%$Pb compared to Cu. This aligns with the observed difference in the tail region of the diffraction profiles for the two materials, as shown in Figure 3(b). The reduced $M^{*}$ parameter in Cu-1$\%$Pb indicates a more ordered dislocation arrangement, i.e., having a higher dipole character, correlated with plastic deformation. As strain increases and the dipole character of the dislocation structure becomes more pronounced^16^, Burger’s vectors appear in pairs, cancelling out each other’s strain fields. This relationship is defined by $M = R_{e}\sqrt{\rho }$, where $R_{e}$ represents the outer cutoff radius beyond which dislocation strain fields are presumed non-interacting^30^. In addition to the dislocation density ($\rho$), the arrangement of the dislocations also directly correlates with the stored elastic energy density within the material^31^. An arrangement with a pronounced dipole character, i.e., a lower value of $R_{e}$ and $M^{*}$, signifies a lower stored energy for the same density.

Given that the nature of a dislocation influences its interactions and annihilation^9,32^, the ratio of edge to screw dislocations was computed as a function of strain for both materials after reaching a 15$\%$ true strain, using the fitted dislocation character value (*q*). Previous research on fine-grained 99.9$\%$ Cu compressed at low temperatures by Schafler et al.^32^ highlighted an uptick in the edge dislocation fraction with strain, culminating in an edge fraction approaching 75$\%$ at a true strain of 22$\%$ before plateauing. Generally, a similar trend favoring the increase in the net generation of edge dislocations with increasing strain is observed for both Cu and Cu-1$\%$Pb. However, the oxygen-free high thermal conductivity (OFHC) Cu, characterized by larger grains (averaging $\sim$ 60 $\mu$m), showed a reversal in net edge versus screw dislocations generated past 30$\%$ strain. This observed shift may primarily be attributed to noise, evident as pronounced fluctuations in the tail region of the diffraction line profiles, making it challenging for eCMWP to accurately decouple dislocation character and arrangement.

Compared to neutron diffraction measurements, which provide an indirect yet quantitative approach to microstructural characterization, electron microscopy techniques like Electron Backscatter Diffraction (EBSD) and Transmission Electron Microscopy (TEM) offer direct insights into local microstructures, dislocation cell structures, and arrangements^33^. The synergy of these methods can enhance our understanding of microstructural evolution inferred from neutron diffraction^15,34^.

**Fig. 4:**
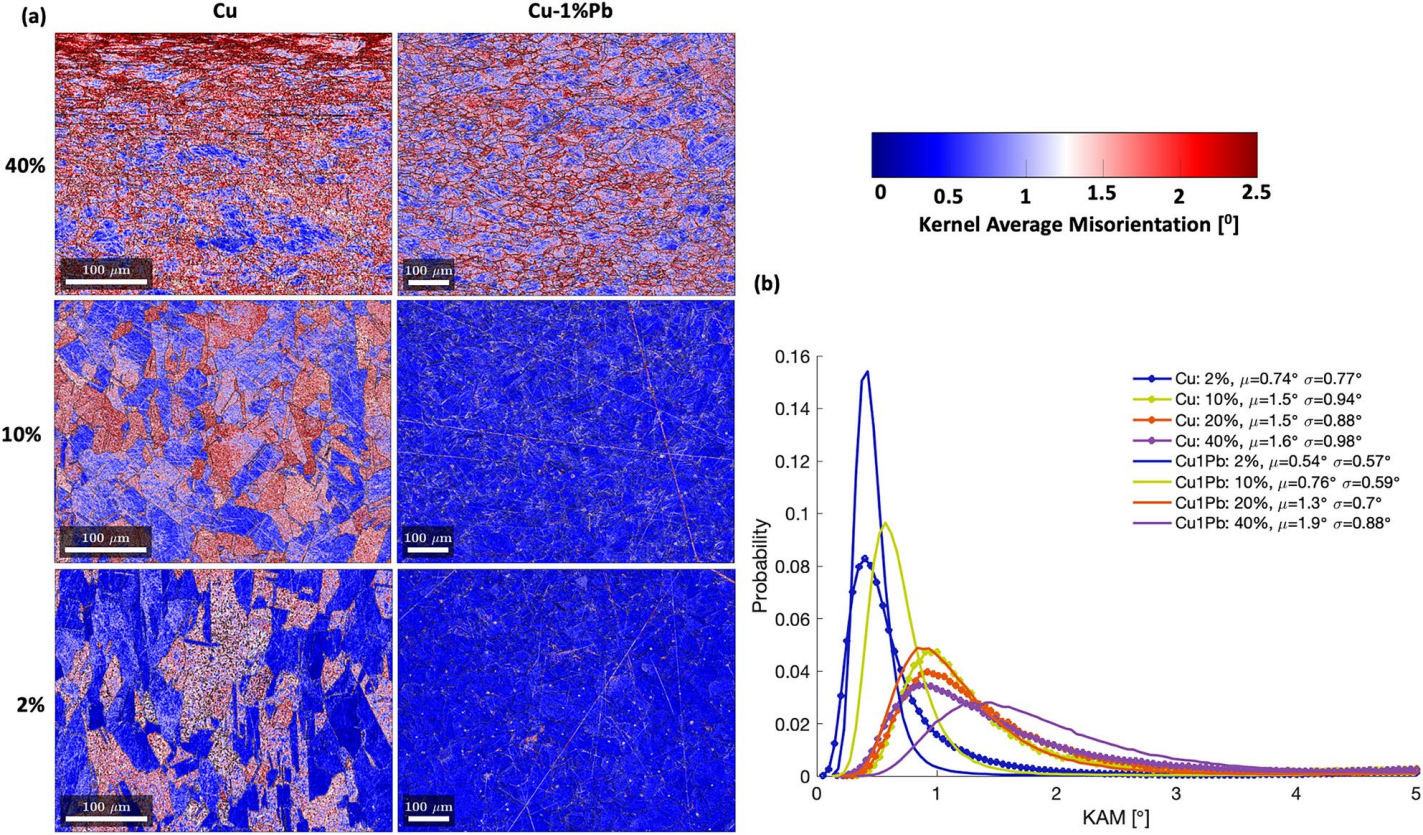
(**a**) KAM maps of deformed microstructures at different strain levels for Cu and Cu-$1\%$Pb. (**b**) Probability distribution of KAM values at different strain levels for the two materials.

Figure 4 shows the Kernel Average Misorientation (KAM) maps of deformed microstructures at varying strains for Cu and Cu-$1\%$Pb. The local grain morphology and orientation changes pre- and post-deformation were characterized using EBSD, and the KAM and the distance between a pixel and its nearest grain boundary were computed using the Dream.3D software^35^. Cu displays KAM accumulation within the grain interior, a phenomenon absent in Cu-1$\%$Pb, particularly at lower strains. Moreover, Cu reveals a crystallographic orientation-dependent deformation accumulation within the grain interior, leading to pronounced orientation gradients in certain grains, while Cu-1$\%$Pb does not exhibit this crystallographic orientation dependency. Instead, elevated KAM values are concentrated near the grain boundary in Cu-1$\%$Pb. Figure 4(b) shows the probability distribution of KAM values across different strain levels for both materials. Although both materials show a rise in mean KAM values with deformation, Cu-1$\%$Pb consistently exhibits lower mean KAM values at lower strains. Interestingly, Cu-1$\%$Pb, which initially displayed high KAM values predominantly near the grain boundary at smaller strains, began to develop an orientation gradient within the grain interior at larger strains, resembling the deformation pattern seen in Cu at equivalent strain levels.

EBSD and DLPA provide complementary but distinct microstructural information. EBSD, with a 0.5 $\mu$m resolution, cannot directly detect sub-grains below this size. The KAM values from EBSD reflect larger scale misorientations and inferred GND densities, which may not correlate with sub-grain sizes detected by DLPA. Cu-1$\%$Pb exhibits consistently smaller $\chi$ values than Cu, especially at higher strain levels (past 5$\%$ strain), indicating finer coherent scattering domains throughout deformation. This refinement is attributed to Pb particles impeding dislocation motion and promoting smaller subgrain formation. These observations are consistent with the higher dislocation density and more homogeneous strain distribution measured in Cu-1$\%$Pb. This observation supports the dual role of Pb particles as dislocation sources and obstacles, resulting in a more refined dislocation substructure in Cu-1$\%$Pb compared to pure Cu during plastic deformation.

**Fig. 5:**
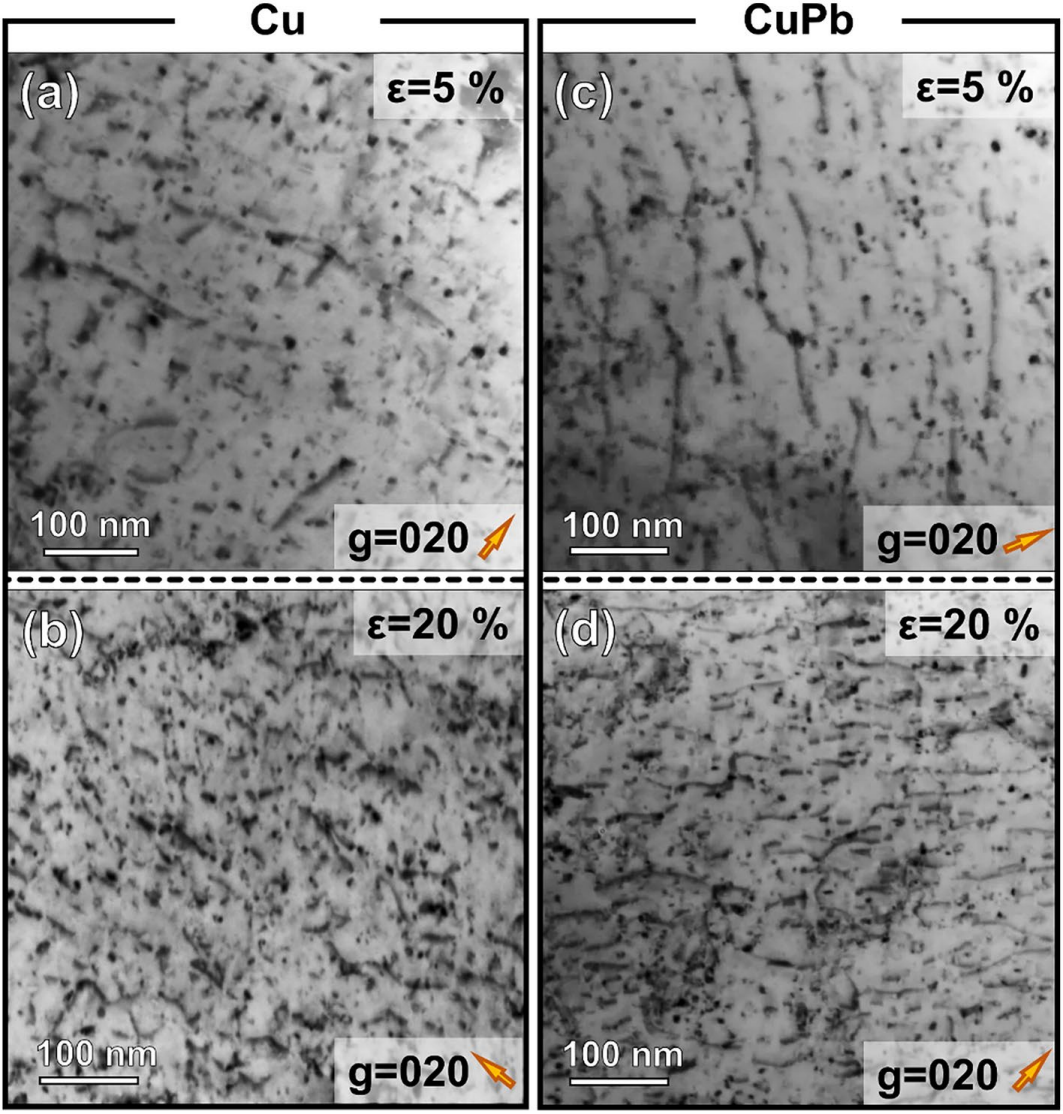
Comparison of dislocation structures in Cu and Cu1$\%$Pb at different strain levels. Bright field (BF) TEM micrographs of Cu deformed to (**a**) 5$\%$ and (**b**) 20$\%$ strain show an increase in dislocation density with strain. In contrast, BF TEM micrographs of Cu1$\%$Pb at (**c**) 5$\%$ and (**d**) 20$\%$ strain indicate a higher dislocation density than that in Cu at equivalent strain levels. All TEM micrographs were captured under two-beam conditions with $\hbox {g}_{020}$ strongly excited, as indicated by the orange arrows in the lower right corners of the micrographs.

Figure 5 shows a comparison of the dislocation structures in both Cu and Cu-1$\%$Pb at 5$\%$ and 20$\%$ strains. All bright-field TEM micrographs were captured under consistent two-beam conditions, with the $\hbox {g}_{020}$ direction being strongly excited. After 5$\%$ deformation, elongated, isolated dislocations are observed in both Cu and Cu-1$\%$Pb (Figures 5(a,c)). Upon deformation to 20$\%$ strain, there is a significant increase in dislocation density (Figures 5(b,d)). The dislocation densities were estimated to be 0.97±0.18 $\times 10^{14} m^{-2}$ and 3.75±0.75 $\times 10^{14} m^{-2}$ for Cu, and 1.35±0.21 $\times 10^{14} m^{-2}$ and 5.99±1.08 $\times 10^{14} m^{-2}$ for Cu-1$\%$Pb, at 5$\%$ and 20$\%$ compressive strain, respectively. The elevated dislocation densities observed in Cu-1$\%$Pb, relative to Cu at identical strain levels, are consistent with the results from the neutron diffraction measurements.

## Discussion

The presence of Pb precipitates significantly influences the movement and interaction of dislocations within the material. Both TEM and DLPA consistently indicated increased dislocation generation due to the addition of a small quantity of Pb to the Cu matrix. To fully appreciate these results, it is crucial to understand the distinctions between dislocation detection capabilities of different characterization techniques employed in this study.

DLPA is sensitive to all dislocations in the material, including both geometrically necessary dislocations (GNDs) and statistically stored dislocations (SSDs). In contrast, EBSD is primarily sensitive to GNDs, inferring a minimum dislocation density necessary to create the pixel-by-pixel misorientation detected. The GND values measured by EBSD are inherently dependent on the spot size used for orientation measurement, which corresponds to the pixel size in KAM maps. Consequently, EBSD only captures a subset of the total dislocation density, while many dislocations, particularly SSDs, remain invisible to EBSD but are detectable by DLPA. This fundamental difference in detection capabilities explains the higher dislocation densities revealed by DLPA compared to those inferred from EBSD measurements, especially in materials with a significant proportion of SSDs, such as the Cu-1$\%$Pb samples.

Typically, the intrinsic excess volume associated with the grain boundary (GB) region promotes dislocation nucleation^10^, allowing these dislocations to propagate within the grain interior during deformation. Scanning electron microscopy of the Cu-1$\%$Pb sample revealed that initially, Pb particles were predominantly segregated at the grain boundaries^6,8^. The presence of Pb precipitates at GB can modify the local stress field, thereby increasing the propensity for dislocation nucleation and enhancing plastic deformation^8^. This effect is accentuated due to the substantial size mismatch between Cu and Pb atoms. Consequently, the presence of Pb precipitates might also modify the energy landscape for dislocation interactions with the grain boundary^9,36^.

While the TEM analysis, specifically the g$\cdot$b analysis presented in Figure 10 of the Additional information section, revealed dislocations with different Burgers vectors under various g-vector conditions in the Cu-1$\%$Pb specimen at 20$\%$ strain, it is challenging to definitively conclude whether more slip systems were activated compared to pure Cu. This limitation arises from the strong orientation dependence of dislocation slip activation and the random selection of grains for TEM characterization, which may not provide a fully representative picture of the material’s bulk behavior. Although both TEM and DLPA techniques provided valuable insights, the characterization of specific active slip systems and their evolution in Cu versus Cu-1$\%$Pb, and their correlation with the observed dislocation density evolution, was not possible within the scope of this study.

To interpret the experimental results, simple finite element unit cell simulations^37^ were performed. The model parameters were calibrated using the experimentally measured macroscopic stress-strain data. Although the small addition of Pb didn’t significantly affect the overall predicted response during compression, it markedly changed the local deformation fields, leading to distinct differences in the local plastic strain development between Cu and Cu-1$\%$Pb.

**Fig. 6:**
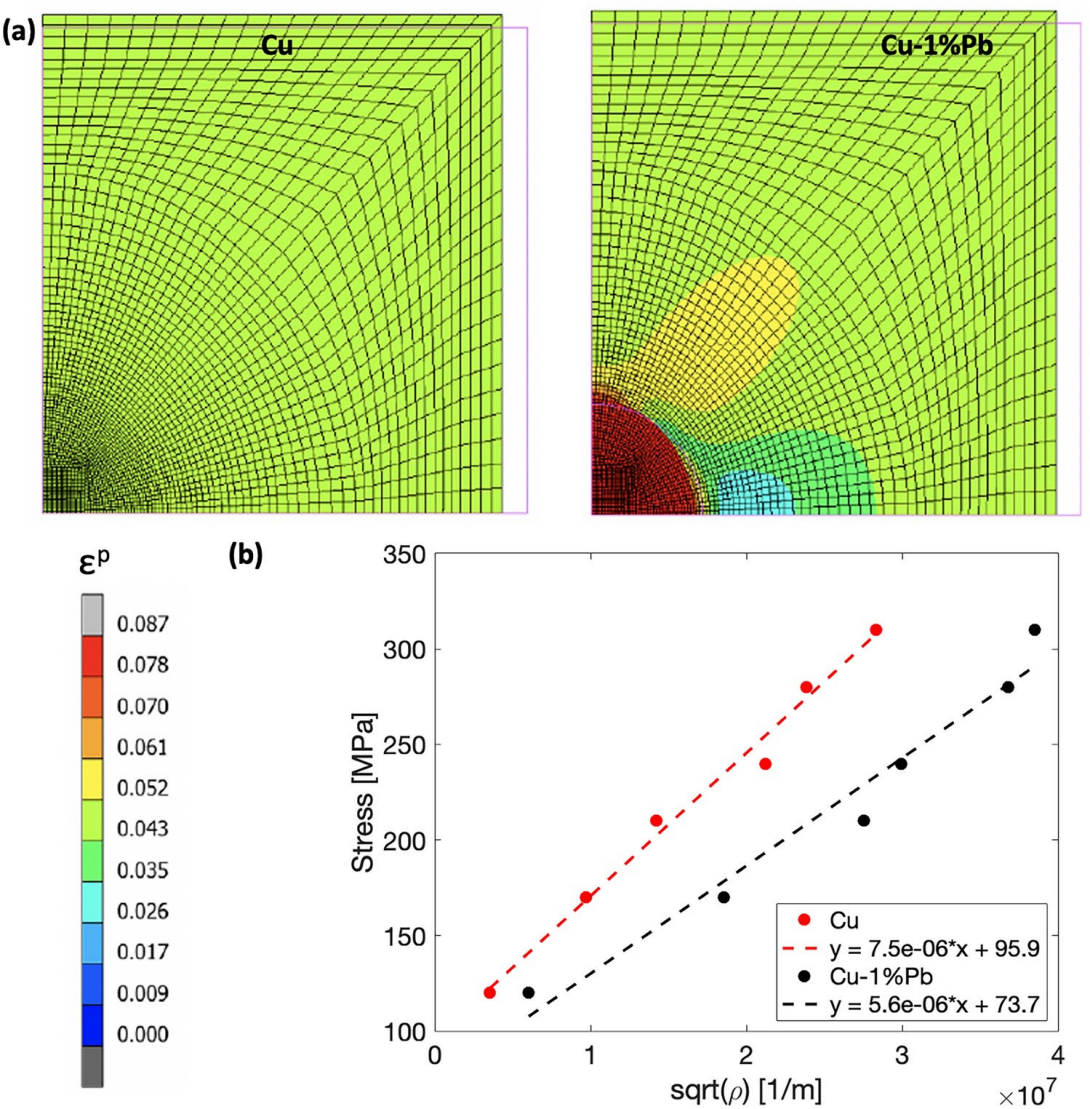
(**a**) Plastic strain maps at 5$\%$ of macroscopic deformation for OFHC Cu (left) and Cu-1$\%$Pb (right). Magenta lines indicate the profile of the undeformed cell. (**b**) Taylor plot showing flow stress versus dislocation density in the two materials.

Figure 6(a) shows the predicted equivalent plastic strain maps at 5$\%$ strain for both Cu (left) and Cu-1$\%$Pb (right). The magenta lines outline the profile of the undeformed cell. In Cu, deformation was observed to be uniform, consistent with the applied macroscopic value. Conversely, for the Cu-1$\%$Pb composite, the introduction of lead localized the plastic strain at the interface between the two materials. This localized strain surpassed the applied macroscopic strain, resulting in a more heterogeneous strain distribution. Beyond the interface area, the remaining copper matrix exhibited a strain distribution that was more uniform and aligned with the macroscopic value.

Although the employed model does not inherently promote the localization of plastic strain, the deformation map for Cu-1$\%$Pb exhibited a distinct behavior due to the presence of Pb precipitates, leading to an anisotropic plastic strain distribution. This can be attributed to the propensity of Pb to locally accumulate deformation more than copper, given its significantly lower elastic modulus and work-hardening rate. Furthermore, it is possible that dislocations are either annihilated or pinned at the Pb particles, primarily located near grain boundaries. This would mean the additional dislocations are not contributing to strength or hardening, potentially explaining the similar macroscale response between the two materials. Therefore, the similar macroscopic stress-strain behavior of Cu and Cu-1$\%$Pb, despite their differences in microstructural evolution, suggests that while Pb particles influence local deformation mechanisms, the overall mechanical response remains primarily controlled by the Cu matrix.

Figure 6(b) shows the flow stress as a function of the square root of the observed dislocation density evolution across strain levels. Plotting in this manner collapses the data into a straight line represented by the Taylor equation:

2 $$\begin{aligned} \sigma _{f} = \sigma _{c}+T\alpha \mu b \sqrt{\rho }, \end{aligned}$$

where $\sigma _{f}$ is the flow stress, $\sigma _{c}$ is an empirical constant, T is the Taylor factor, $\alpha$ is the strength of the obstacles that a moving dislocation has to overcome during deformation, $\mu$ is the shear modulus, and b is the Burger’s vector for slip system <111> $\{$110$\}$^38^.

For both materials, an increase in flow strength corresponds with a rise in dislocation density during deformation. However, in Cu-1$\%$Pb, more dislocations are generated to achieve the same hardening level as in pure Cu. Based on both experimental observations and simulations, it appears that stress builds up around the Pb precipitate located near grain boundaries, thereby pinning dislocations at lower strains. As strain increases, the influence of Pb precipitates on dislocation movement diminishes, allowing dislocations to move within the grain interior. This results in a similar pattern of orientation gradient development as observed in Cu at larger strains. Further evidence for this shift in deformation mechanisms is the observed slowdown in the rate of dislocation density increase for Cu-1$\%$Pb after 30$\%$ strain.

In summary, this work provides a comprehensive study of the microstructural evolution and plastic deformation behavior of Cu and Cu-1$\%$Pb. The combination of advanced characterization techniques, including DLPA, EBSD, TEM, and finite element simulations, revealed that the presence of lead precipitates, primarily located near grain boundaries, significantly affects the local plastic deformation during compression, despite not altering the macroscopic texture evolution and stress-strain response compared to pure Cu. The findings highlight the complex interplay between alloying elements, microstructural features, and deformation mechanisms. The Pb precipitates modify the local stress field, increase the propensity for dislocation nucleation, and affect the energy landscape for dislocation interactions with grain boundaries. As strain increases, the influence of these precipitates diminishes, allowing dislocations to move within the grain interior, resembling the deformation behavior of pure copper. The insights gained from this study can be valuable in tailoring the properties of structural materials for specific applications, as they provide a deeper understanding of the structure-property relationships in immiscible alloy systems.

However, despite our comprehensive approach, characterizing the orientation relationship between the copper and lead phases and its evolution with strain proved challenging. The small size of the Pb particles (1-5 microns) resulted in a high surface-to-volume ratio, likely causing surface atoms to be displaced from their regular lattice positions. This displacement contributes to diffuse scattering rather than Bragg-type scattering, explaining the absence of a detectable Pb phase signal in both texture and DLPA measurements^39^. Advanced methods such as the core-shell model proposed by Palosz et al.^39^ for nanocrystals could potentially offer insights into the local behavior of the Cu-1$\%$Pb system. EBSD and TEM techniques were also unable to index the Pb particles due to their small size and the differential milling rates during sample preparation, with Pb particles being removed before the Cu matrix was sufficiently thin for high-quality TEM imaging. Consequently, with the characterization techniques employed in this study, it was not possible to determine the orientation relationship between the two phases or its evolution under strain. This limitation contrasts with our previous work on Cu-10$\%$W systems^40^, where larger W particles ($\sim$10-20 $\mu$m) allowed for successful characterization using high-energy X-ray diffraction microscopy and neutron diffraction, enabling the observation of particle debonding from the Cu matrix during deformation. However, the current challenges in characterizing the Pb phase highlight how particle size significantly affects our ability to quantify stress and strain partitioning between phases.

The interaction between the matrix and second-phase particles in two-phase metallic systems is complex and can significantly influence the material’s mechanical behavior. The particle-matrix interface often represents the most highly stressed region within the matrix^2^. Under sufficient stress, this interface can fail, leading to cavity formation and potential particle fracture^2,40^. While FEM simulations provide valuable initial insights into local-scale differences between materials with and without second-phase particles, they may not fully capture the intricate physics of systems with second-phase particles such as Pb in Cu. For instance, Pb particles can act as sinks for dislocations, a phenomenon that will require more advanced modeling techniques to accurately represent. Advanced approaches such as phase field models and dislocation dynamics are necessary to fully capture the underlying mechanisms^41,42^. These models can account for phenomena like dislocation-particle interactions, interface decohesion, and particle fracture, providing a more comprehensive understanding of the material’s behavior under various loading conditions.

## Methods

### Mechanical testing

Cylindrical compression specimens for Cu, measuring 8 mm in diameter and 19.6 mm in length, and for Cu-1$\%$Pb, measuring 5 mm by 5 mm, were obtained from a rolled plate. After machining, the Cu samples underwent heat treatment at $600^\circ$ C for 1 hour, while the Cu-1$\%$Pb samples were treated at the same temperature for 2 hours. This ensured that both materials started with random textures and comparable grain sizes^6^. However, a weak rolling texture was evident in the initial state of the Cu specimen. These cylindrical specimens were subjected to compressive strain and deformed to various levels, reaching a maximum strain of 40$\%$ (with a strain rate of 1e-3 /s at room temperature). During the compression tests, a MoS2 lubricant was applied to both ends of the specimens. This minimized friction between the sample and the platens, ensuring uniaxial compression without issues like buckling or barreling.

### Neutron diffraction

Ex-situ specimens of both Cu and Cu-1$\%$Pb were prepared by subjecting multiple samples to varying levels of compressive strains under uniaxial stress conditions. The addition of a small amount of Pb to the Cu matrix had a negligible effect on the macroscopic response of the materials. The high-statistics, high-resolution neutron diffraction measurements for diffraction line profile analysis (DLPA) were conducted using the SMARTS diffractometer. The full orientation distribution functions data for texture determination were collected on the HIgh-Pressure Preferred Orientation (HIPPO) diffractometer at the Lujan Center at LANSCE. Details of SMARTS^43^ and HIPPO^44^ are available elsewhere. High-resolution data (FWHM $\sim$0.1$\%$) for dislocation line profile analysis were collected in the backscattering ($153^\circ$) detector bank on SMARTS. Each specimen at a given strain state had a count time of approximately 10 hours for the line profile measurements, ensuring high signal-to-noise data, as significant information resides in the tails of the diffraction peak profiles. The SMARTS data underwent quantitative line profile analysis using the eCMWP software^26^. The full orientation distribution function (ODF) for texture analysis was obtained from the HIPPO data using the MAUD software package^45^, with detailed procedures described in Wenk et al.^46^.

### Microstructure characterization

#### Electron backscatter diffraction

The samples were sectioned along their longitudinal axis and set in cold mounting resin to inspect the central plane. The sample preparation involved grinding with SiC paper of increasing fineness (from 500 to 2400 grit), followed by mechanical polishing using 1 $\mu$m alpha alumina slurry and 0.04 $\mu$m colloidal silica suspension. A Zeiss Axio ImagerM2m optical microscope captured the deformation profile post-testing. The samples were then etched using a solution of 40ml H2O, 10 ml HCL, and 5 grams of ferric chloride for roughly 10 seconds. After etching, the samples were imaged again with the optical microscope to observe the microstructural changes between the initial and deformed states. The samples underwent another round of polishing with 0.04 $\mu$m colloidal silica suspension and a brief 3-second etch before EBSD imaging. A Thermo Fisher Scientific Apreo SEM microscope, in conjunction with the EDAX OIM Analysis$^{\textrm{TM}}$ software suite, was employed to gather crystallographic orientation and texture data. EBSD scans were conducted with a 0.5 $\mu$m step size.

#### Transmission electron microscopy

For transmission electron microscopy (TEM) analysis, foils were extracted from the surfaces of deformed specimens using an FEI Helios 600 dual beam focused ion beam (FIB)/SEM. Each TEM lamella was thinned down to approximately 200 nm using a 0.3 nA beam current and 30 keV voltage. This was followed by a final polish with a 2 keV beam at a 27 pA current until the lamella became electron transparent. The dislocation structures across various deformation levels in Cu and Cu-1$\%$Pb specimens were captured using an FEI Titan 80-300 equipped with a monochromator and image aberration corrector, operating at 300 kV. The dislocation structures in both materials at different strain levels were examined using two-beam kinematical TEM techniques. To quantify and compare the dislocation densities in Cu and Cu-1$\%$Pb at identical strain levels, the TEM foils were tilted near the [001] zone axis with either $\hbox {g}{020}$ or $\hbox {g}{200}$ being strongly excited. The Burgers vector of the dislocations was determined using the $g\cdot b$ invisibility criterion.

### Micromechanical simulations

A cylindrical elasto-plastic Cu-matrix with an embedded elasto-plastic spherical Pb-inclusion was modeled in the finite element code MSC Marc v2022^47^. Axisymmetric analyses were conducted under large displacement and finite strain conditions, utilizing a Lagrangian update procedure. The matrix and the embedded particle were assumed as perfectly bonded, and a glue contact was used to ensure the continuity of the displacement field^48^. A compressive load was applied imposing a fixed axial displacement on the master node at the cell upright corner. Additionally, plane-sections-remain-plane boundary conditions were implemented, tying the displacement of the associated nodes to the master node through tying equations.

Both the Cu matrix and the Pb particle were modeled as elastic-plastic materials, with a linear and isotropic elastic behavior, described by Young’s modulus *E* and Poisson’s ratio $\nu$, and an isotropic Von-Mises hardening. The hardening rule parameters for both materials were determined by fitting the results of compressive uniaxial true-stress versus true-strain tests conducted at room temperature with an approximate deformation rate of $10^{-3}$/s. The plastic behavior of the Cu matrix was modeled with a two-term Voce hardening law, in which the stress level $\sigma$ is linked to the equivalent plastic deformation $\epsilon _{p}$ as:

$$\begin{aligned} \sigma = \sigma _{0}^{Cu}+\sum _{i=1}^{2}{Q_{i}\left( 1-exp\left( \frac{-\epsilon _{p}}{t_{i}}\right) \right) }, \end{aligned}$$

where $\sigma _{0}^{Cu}$ is the yield stress of Cu and ${Q_{i}}$ and ${t_{i}}$ are the hardening saturation coefficient and exponent, respectively. The mechanical behavior of the Pb particle was evaluated from the data reported in^49^ and fitted with power law relation given by:

$$\begin{aligned} \sigma = \sigma _{0}^{Pb}+A\epsilon _{p}^{n}, \end{aligned}$$

where $\sigma _{0}^{Pb}$ is the yield stress of Pb and *A* and *n* are the hardening coefficient and exponent, respectively.

## Additional information

**Fig. 7:**
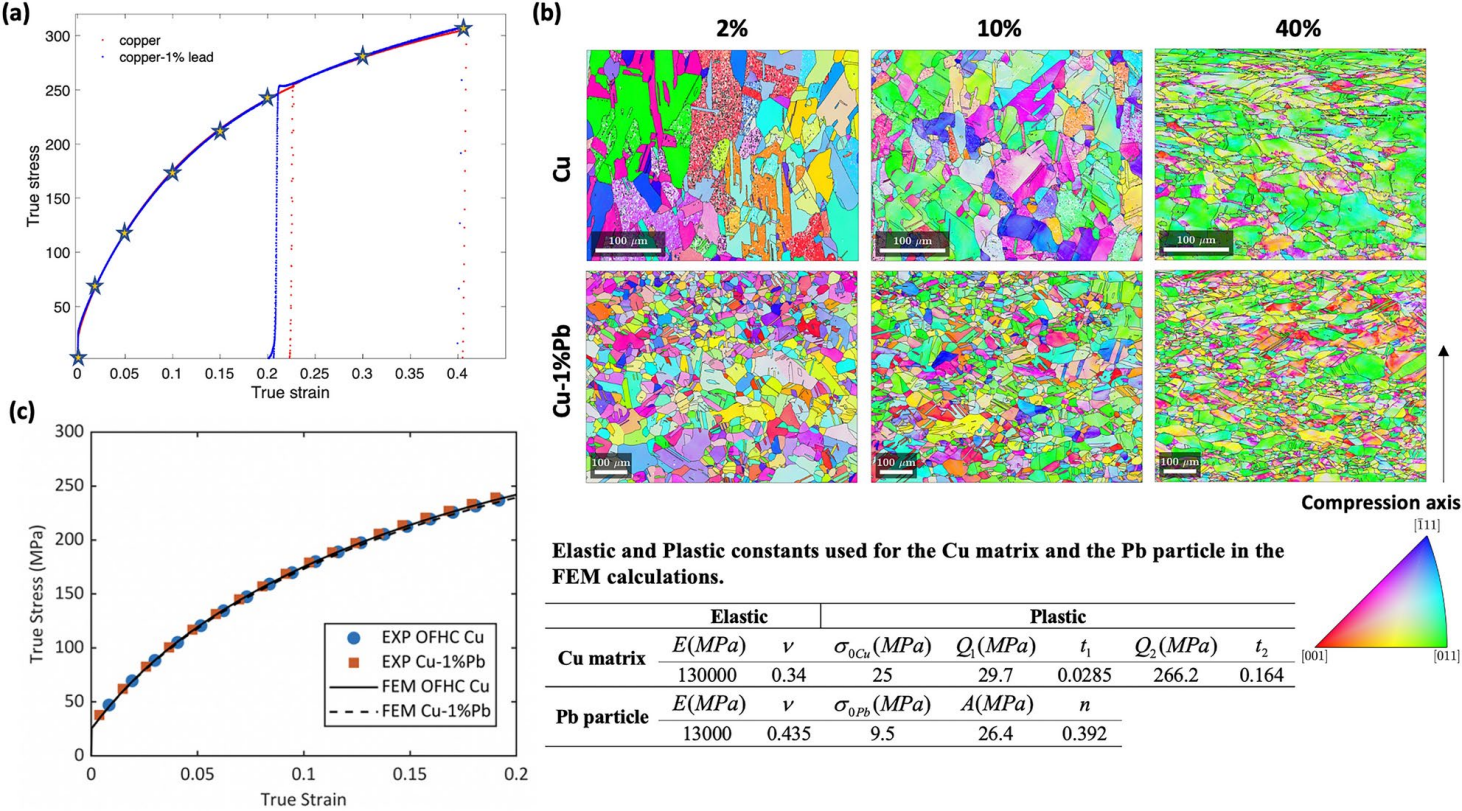
(**a**) Macroscopic stress strain response of Cu and Cu-1$\%$Pb specimens under compressive loading. The star denotes the location where ex situ neutron diffraction data were collected.(**b**) EBSD characterization showing crystallographic orientation maps of deformed microstructures at different strain levels for Cu (top) and Cu-$1\%$Pb (bottom).(**c**) Comparison of experimental true stress and true strain curves with the homogeneous response of the unit cell.

Figure 7(a) shows the macroscopic stress and strain response of the Cu and Cu-1$\%$Pb specimens at different strain levels. The star symbol indicates the strain levels at which neutron diffraction measurements were recorded for both materials. Figure 7(b) shows crystal orientation maps for both materials at different deformation levels. The material constants used in the simulations and the comparison of the predicted stress strain curve using the homogeneous response of the unit cell with the experiment are shown in Figure 7(c).

**Fig. 8:**
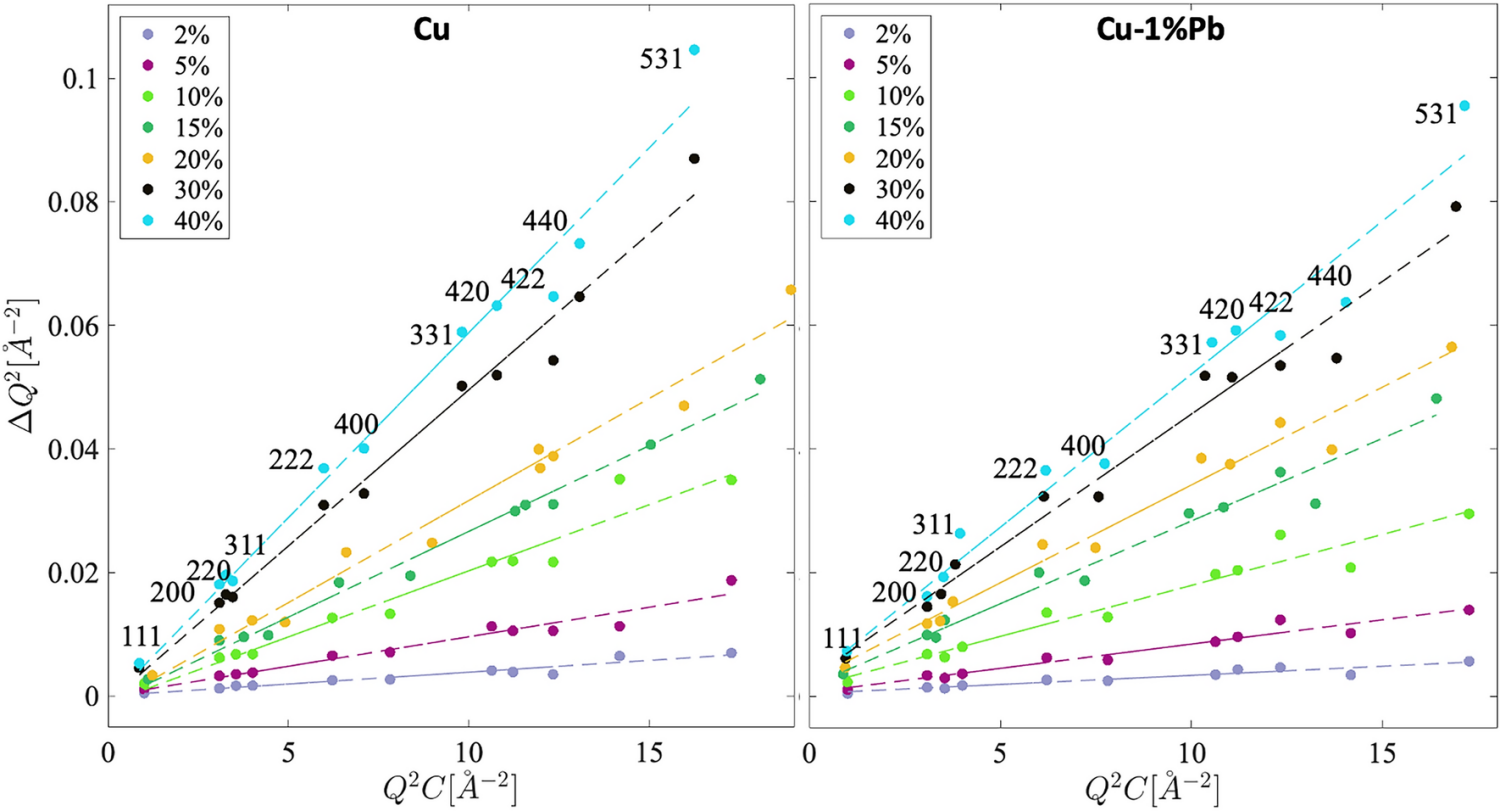
Modified Williamson-Hall plots for copper and copper 1 wt.$\%$ lead. By accounting for the dislocation contrast factor, a linear increase in peak broadening with crystallographic hkl planes is evident.

Figure 8 shows the modified Williamson-Hall (WH) plot. Here the width ($\Delta$Q) of the peaks exhibits anisotropy as a function of the scattering vector (Q), with a consistent increase observed for higher order peaks (e.g. 111, 222, etc.). Strain anisotropy, the non-monotonous dependence of peak broadening as a function of scattering vector, evident in Figure 3(a), is accounted for by the average contrast factors for individual diffraction peaks. Using the elastic constants of Cu, parameters $\hbox {C}_{h00}$ = 0.255^24^ and *q* values (as given in Table 1) were chosen for the Cu and Cu-1$\%$Pb materials to calculate the contrast factor for each hkl reflection. Properly accounting for the dislocation contrast factor in the modified WH plot collapses the peak width data on to a line, confirming that the generation of dislocations during deformation caused the observed anisotropic broadening of diffraction peak profiles in both materials. However, it is not recommended to estimate dislocation density directly from the modified WH plot for reliable results.

**Figure 9:**
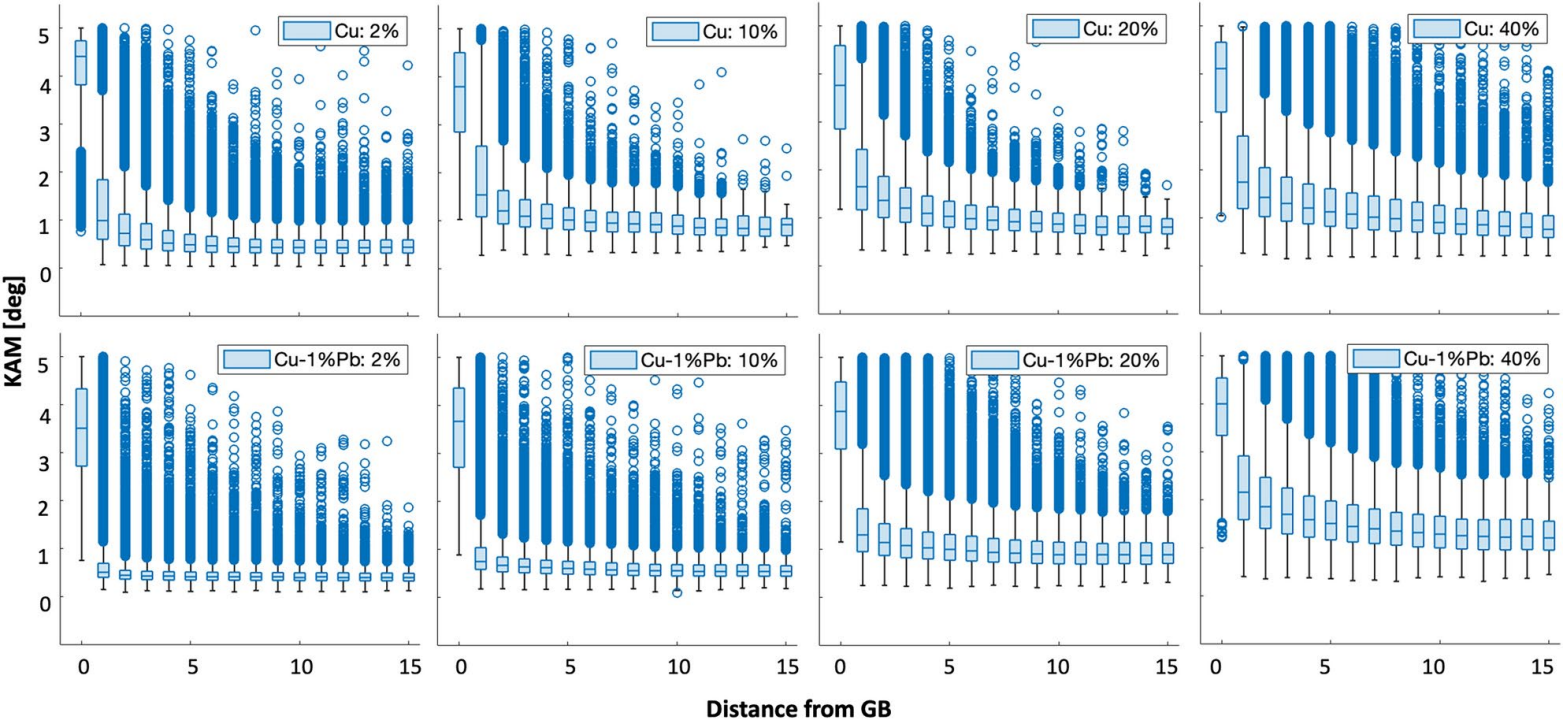
Box plots illustrating the relationship between KAM and distance from the nearest grain boundary at various strain levels for Cu and Cu-$1\%$Pb.

Figure 9(a) shows box plots of KAM values against their distance from grain boundaries for Cu and Cu-$1\%$Pb. The median KAM values for each distance are represented by the line inside the box. The bottom and top edges of the box depict the lower and upper quartiles, respectively. Whiskers extend from the box to the lowest and highest values in the data, excluding outliers (calculated using the interquartile range). Outliers are denoted by open circles beyond the range of the whiskers. Generally, both materials exhibit higher KAM values near the grain boundaries and lower values within the grain interior.

**Fig. 10:**
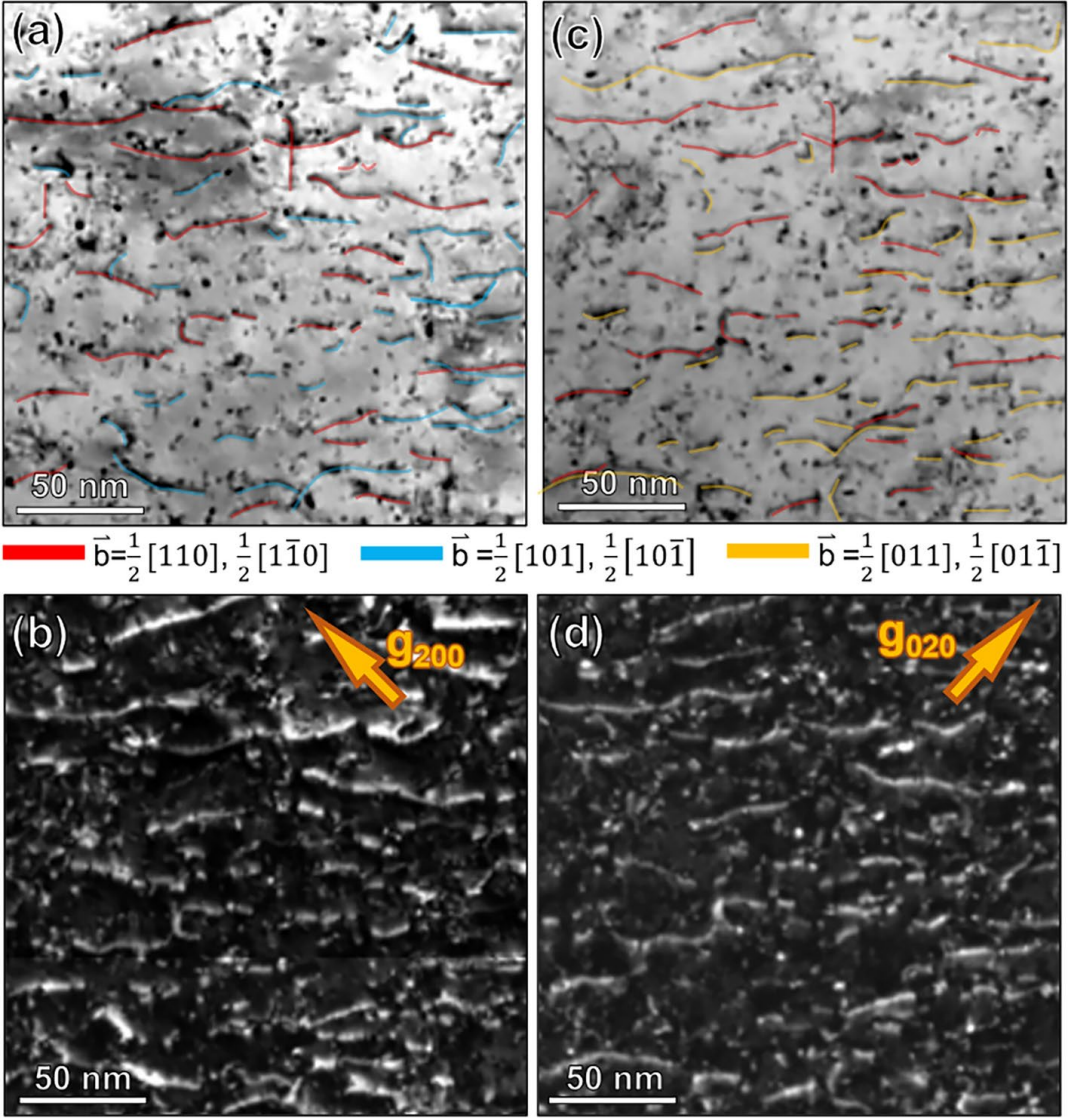
Bright field (BF) and corresponding dark field (DF) TEM micrographs showing the dislocation structures in Cu-1$\%$Pb at 20$\%$ strain. Panels (**a**-**b**) have $\hbox {g}{200}$ strongly excited, while (**c**-**d**) have $\hbox {g}{020}$. Dislocations with different Burgers vectors are color-coded: b= 1/2 [110] and b= 1/2[1-10] in red, b=1/2[101] and b= 1/2[10-1] in blue, b= 1/2[011] and b= 1/2[01-1] in yellow.

Figure 10 shows the two-beam kinematical bright field (BF) and dark field (DF) TEM images of Cu-1$\%$Pb deformed to a 20$\%$ compressive strain, captured in the same regions. Dislocations with distinct Burgers vectors are highlighted in different colors in Figures 10(a,c): b= 1/2 [110] and b= 1/2[1-10] are in red, b=1/2[101] and b= 1/2[10-1] are in blue, and b= 1/2[011] and b= 1/2[01-1] are in yellow.

## Data Availability

The data will be made available upon reasonable request to the corresponding author (reeju@lanl.gov).
